# Dr. Thomas Brown Wheeler

**Published:** 1901-02

**Authors:** 


### OBITUARY NOTES.

Dr. Thomas Brown Wheeler, of Montreal, died suddenly at the Murray Hill Hotel, New York, on January 10, 1901, of apoplexy. He was near 70 years of age.

